# Tryptophan and Its Selected Metabolites Modulate Melanoma-Cell Biology: A Preliminary In Vitro Study

**DOI:** 10.3390/ijms27219908

**Published:** 2026-11-05

**Authors:** Anna Michalak-Stoma, Katarzyna Walczak, Małgorzata Kowal, Katarzyna Sawa-Wejksza, Michał Chojnacki, Marta Kinga Lemieszek, Filip Stoma, Dorota Krasowska

**Affiliations:** 1 Chair and Department of Dermatology, Venereology and Paediatric Dermatology, Medical University of Lublin, Staszica 11, 20-081 Lublin, Poland; dorota.krasowska@umlub.edu.pl; 2 Laboratory for Immunology of Skin Diseases, Chair and Department of Dermatology, Venereology and Paediatric Dermatology, Medical University of Lublin, Radziwiłłowska 11, 20-080 Lublin, Poland; katarzyna.walczak@umlub.edu.pl (K.W.);; 3 Department of Virology and Immunology, Maria Curie-Sklodowska University, Akademicka 19, 20-033 Lublin, Poland; katarzyna.sawa-wejksza@umcs.pl; 4 Department of Medical Biology, Institute of Rural Health, Jaczewskiego 2, 20-090 Lublin, Poland; chojnacki.michal@imw.lublin.pl (M.C.); lemieszek.marta@imw.lublin.pl (M.K.L.); 5 Chair and Department of Neurosurgery, Medical University of Lublin, Jaczewskiego 8, 20-090 Lublin, Poland; filip.stoma@umlub.edu.pl

**Keywords:** melanoma, tryptophan, tryptophan metabolism, kynurenine pathway, anthranilic acid, cinnabarinic acid, vemurafenib, BRAF, cell cycle, tumor–immune interactions

## Abstract

Tryptophan (TRP) and its metabolites are involved in numerous physiological and pathological processes within the skin. They may influence melanoma biology, but their role remains insufficiently understood. This study investigated the effects of TRP, anthranilic acid (AA), and cinnabarinic acid (CBA) in melanoma cells in vitro and their potential interaction with the B-Raf proto-oncogene, serine/threonine kinase (BRAF) inhibitor vemurafenib. A375, G361, and SK-MEL-3 melanoma cell lines and a THP-1-derived macrophage model were used. Cell proliferation and metabolic activity were assessed using the 3-(4,5-dimethylthiazol-2-yl)-2,5-diphenyltetrazolium bromide (MTT) assay, migration using a scratch assay, and cell-cycle distribution by flow cytometry. Western blotting was used to analyze selected signaling proteins. Reactive oxygen species (ROS) and nitric oxide (NO) production were evaluated. Bioinformatic analyses investigated associations between *BRAF* and genes related to tryptophan metabolism and the kynurenine pathway. All compounds showed dose-dependent antiproliferative effects, with prolonged exposure increasing efficacy. TRP and AA increased G0/G1 cell-cycle arrest, whereas CBA increased the G2/M population. TRP also reduced A375 cell migration. All three compounds enhanced the antiproliferative effect of vemurafenib, with CBA showing the strongest effect (approximately 20%). Western blot analysis indicated modulation of p21 expression and ERK1/2 phosphorylation, suggesting implications for cell cycle and stress signaling pathways. Moreover, conditioned medium from A375 cells exposed to tested compounds affected cell viability, ROS, and NO production in THP-1-derived macrophages. TRP and its metabolites may be considered potential supportive agents for melanoma BRAF-targeted therapy. However, further in vivo studies are needed to clarify their therapeutic value and to elucidate the underlying molecular mechanisms.

## 1. Introduction

The role of tryptophan (TRP) and its biologically active metabolites in physiological and pathological processes within the skin is not fully understood [1,2,3,4,5,6]. The skin is constantly exposed to endogenous and exogenous sources of TRP derivatives. The stratum corneum contains amino acids originating from keratin and dead keratinocytes, which are believed to serve as substrates for TRP metabolism by the skin microbiota [7]. TRP metabolites are also present in human sweat [8,9] and in herb or plant extracts used in body treatments [10,11]. Approximately 95% of TRP is metabolized via the kynurenine pathway (KP), leading to the synthesis of nicotinamide adenine dinucleotide (NAD) and several biologically active metabolites [12,13]. The role of TRP and its metabolites in carcinogenesis and metastasis is not fully understood, although research in this area has mainly focused on immune regulation [14,15,16]. However, TRP metabolites may also affect cancer-cell proliferation, survival, or migration [1,3,17].

Melanoma is characterized by high potential for metastasis and intrinsic resistance to anticancer drugs [18,19,20]. One of the groups of melanoma-targeted therapies is B-Raf proto-oncogene, serine/threonine kinase (BRAF) inhibitors, including vemurafenib. *BRAF* mutations are detected in approximately 50% of melanomas. However, the use of BRAF inhibitors as monotherapy may result in the development of melanoma resistance [21]. In addition to BRAF inhibitors, immune checkpoint blockers (ICB) have been approved for the treatment of advanced melanoma [22]. Nevertheless, the response rate to anti-PD1 antibodies generally remains below 40%, suggesting the presence of immune evasion and resistance mechanisms [23]. Elmer et al. revealed a novel immunosuppressive mechanism in melanoma and non-melanoma skin cancers driven by the combined activation of Hedgehog/GLI (HH/GLI) with pro-inflammatory IL6/STAT3 or IFNγ/STAT1 signaling, which together enhance the expression of indoleamine 2,3-dioxygenase 1 (IDO1) [20]. IDO1 upregulation induces immunosuppression, tumor proliferation, invasion, and migration by three key mechanisms: (1) induction of immunosuppressive antigen-presenting cells, (2) depletion of TRP and accumulation of KP metabolites, which activate pathways such as aryl hydrocarbon receptor (AhR), leading to T-cell apoptosis and Th17 suppression, and (3) recruitment of myeloid-derived suppressor cells (MDSCs) that enhance the immunosuppressive tumor microenvironment [20,24,25]. Elevated levels of kynurenine (KYN) have been observed in the serum and tumor microenvironment of melanoma patients and are associated with poor prognosis and resistance to immunotherapy. Thus, targeting the KP is being explored as a therapeutic strategy in melanoma [20,26].

**Figure 1:**
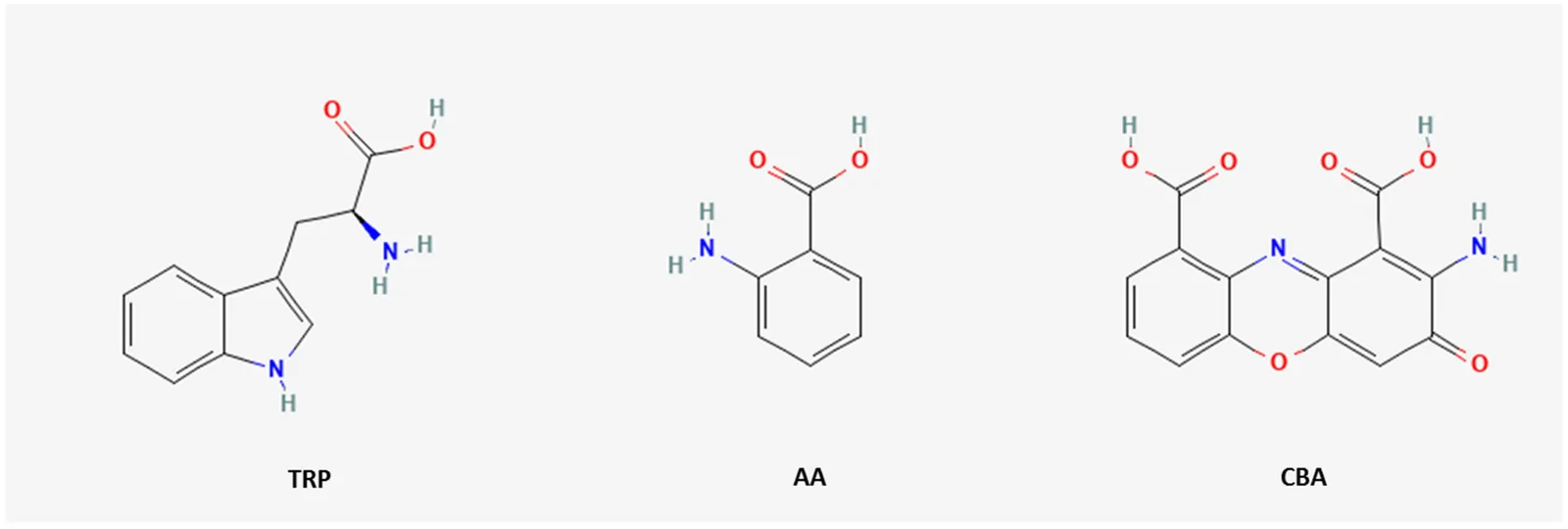
The molecular structure of TRP (2D structure image of CID 6305: PubChem Identifier: CID 6305; https://pubchem.ncbi.nlm.nih.gov/compound/6305#section=2D-Structure URL accessed on 13 October 2026 [27]), AA (2D structure image of CID 227: PubChem Identifier: CID 227, https://pubchem.ncbi.nlm.nih.gov/compound/227#section=2D-Structure URL accessed on 13 October 2026 [28]), and CBA (2D structure image of CID 114918: PubChem Identifier: CID 114918, https://pubchem.ncbi.nlm.nih.gov/compound/114918#section=2D-Structure URL accessed on 13 October 2026 [29]) generated from PubChem [30].

Our previous studies revealed the biological activity of kynurenic acid (KYNA), KYN, and 6-formylindolo [3,2-b]carbazole (FICZ) in melanoma cells in vitro [1]. Therefore, this study aimed to determine whether TRP and its selected metabolites, anthranilic acid (AA) and cinnabarinic acid (CBA) (Figure 1), affect the proliferation, migration, and cell cycle of melanoma cells in vitro. Moreover, we identified the biological interactions between TRP, AA, CBA, and the BRAF inhibitor vemurafenib in an in vitro model of the melanoma–immune cell interaction to verify the potential of TRP and its metabolites as supportive agents in melanoma treatment.

## 2. Results

**Figure 2:**
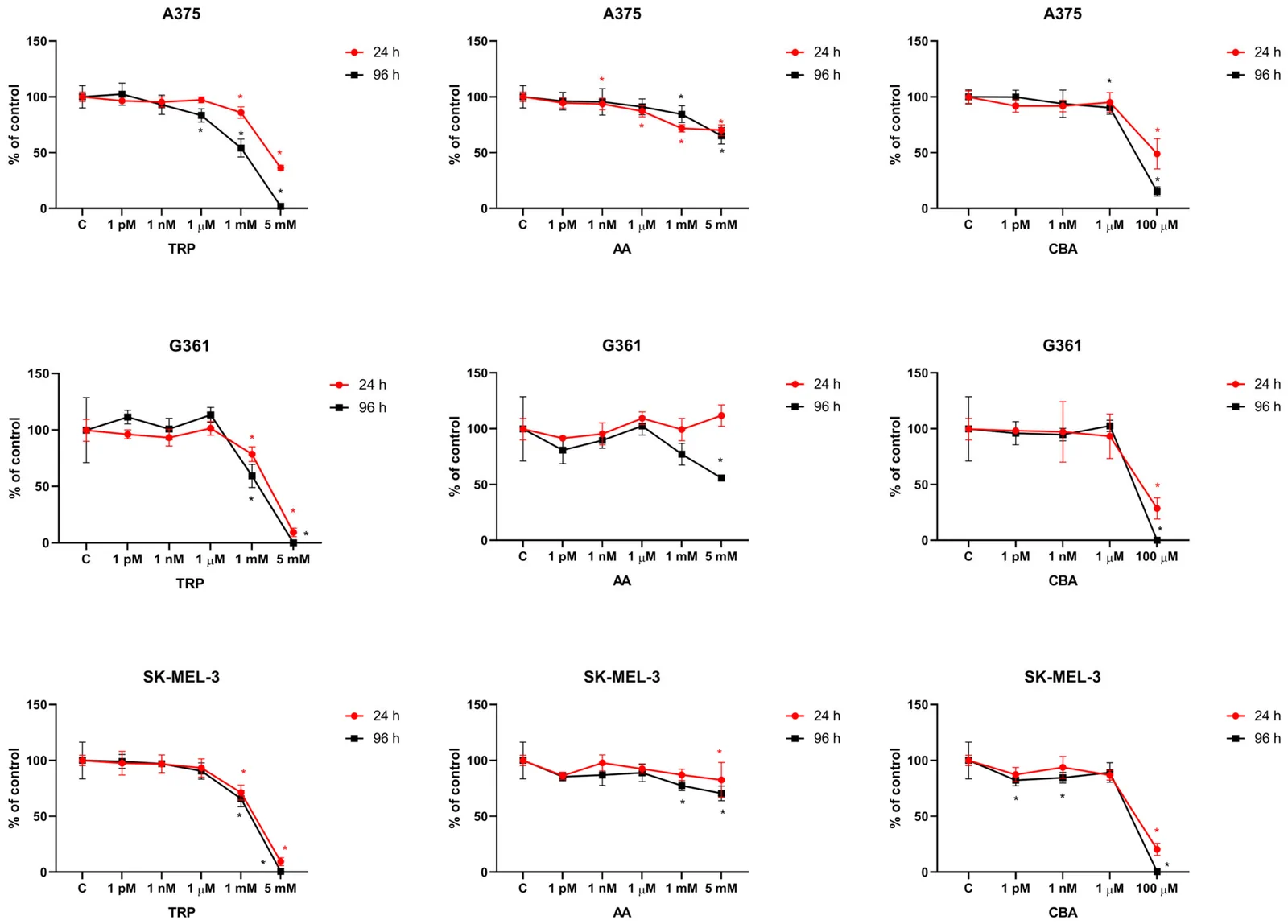
The effect of TRP and its selected metabolites on proliferation and metabolic activity of melanoma cells. A375, G361, and SK-MEL-3 cells were exposed to a range of concentrations of TRP, AA (1 pM, 1 nM, 1 µM, 1 mM, 5 mM), and CBA (1 pM, 1 nM, 1 µM, 100 µM) for 24 and 96 h. The effect of the tested substances on proliferation and metabolic activity was determined using the MTT assay. Data are presented as the mean value (*N* ≥ 4, independent biological replicates) and standard deviation (SD). Data are presented as % of control (Control—untreated, 100%). * statistical significance (one-way ANOVA with Dunnett post hoc test, *p* < 0.05).

The effect of TRP and its selected metabolites, AA and CBA, on proliferation and metabolic activity of melanoma A375, G361, and SK-MEL-3 cells was assessed using the MTT assay (Figure 2). MTT assay results integrate several biological activities, including cell proliferation, viability, metabolic rate, and growth phase [31]. A375 and G361 cells represented primary melanoma, whereas SK-MEL-3 cells represented metastatic melanoma. TRP inhibited proliferation and metabolic activity of all melanoma-cell lines after 24 and 96 h, and the effect was dependent on the concentration and incubation time. TRP at 1 mM and 5 mM inhibited proliferation and metabolic activity of melanoma cells by 14.2% and 63.6% for A375, 21.4% and 90.7% for G361, and 28.8% and 90.6% for SK-MEL-3 after 24-h exposure. The inhibitory effect of TRP increased with longer 96-h incubation. TRP inhibited proliferation and metabolic activity of A375 melanoma cells by 16.6% at 1 µM, 45.9% at 1 mM, and 98% at 5 mM. TRP reduced proliferation of G361 cells by 40.6% at 1 mM and 100% at 5 mM. A similar effect was observed in SK-MEL-3 cells. TRP inhibited proliferation and metabolic activity of SK-MEL-3 cells by 34.3% at 1 mM and 99.3% at 5 mM. AA exhibited the strongest inhibitory activity against A375 cells. AA decreased the proliferation of A375 cells after 24-h incubation by 6.4% at 1 nM, 12.8% at 1 µM, 28.2% at 1 mM, and 29.9% at 5 mM. AA did not affect the proliferation and metabolic activity of G361 cells after 24-h incubation, whereas AA at 5 mM inhibited the proliferation and metabolic activity of SK-MEL-3 cells by 17.5% after 24-h exposure. However, 96-h exposure to AA led to inhibited proliferation and metabolic activity of all tested melanoma cell lines (A375: 1 mM—15.5%, 5 mM—34.8%; G361: 5 mM—44.2%; SK-MEL-3: 1 mM—22.6%, 5 mM—29.5%). The inhibitory activity of CBA at 100 µM was observed in all melanoma-cell lines after 24-h incubation (A375: 51%; G361: 71.4%; SK-MEL-3: 79.6%). The antiproliferative effect of CBA was also time-dependent. CBA inhibited proliferation and metabolic activity of A375 cells by 9.6% at 1 µM and 84.8% at 100 µM. CBA at 100 µM inhibited proliferation and metabolic activity of G361 cells by 99.9%. CBA reduced proliferation and metabolism of SK-MEL-3 cells by 17.7% at 1 pM, 15.4% at 1 nM, and 99.6% at 100 µM.

**Figure 3:**
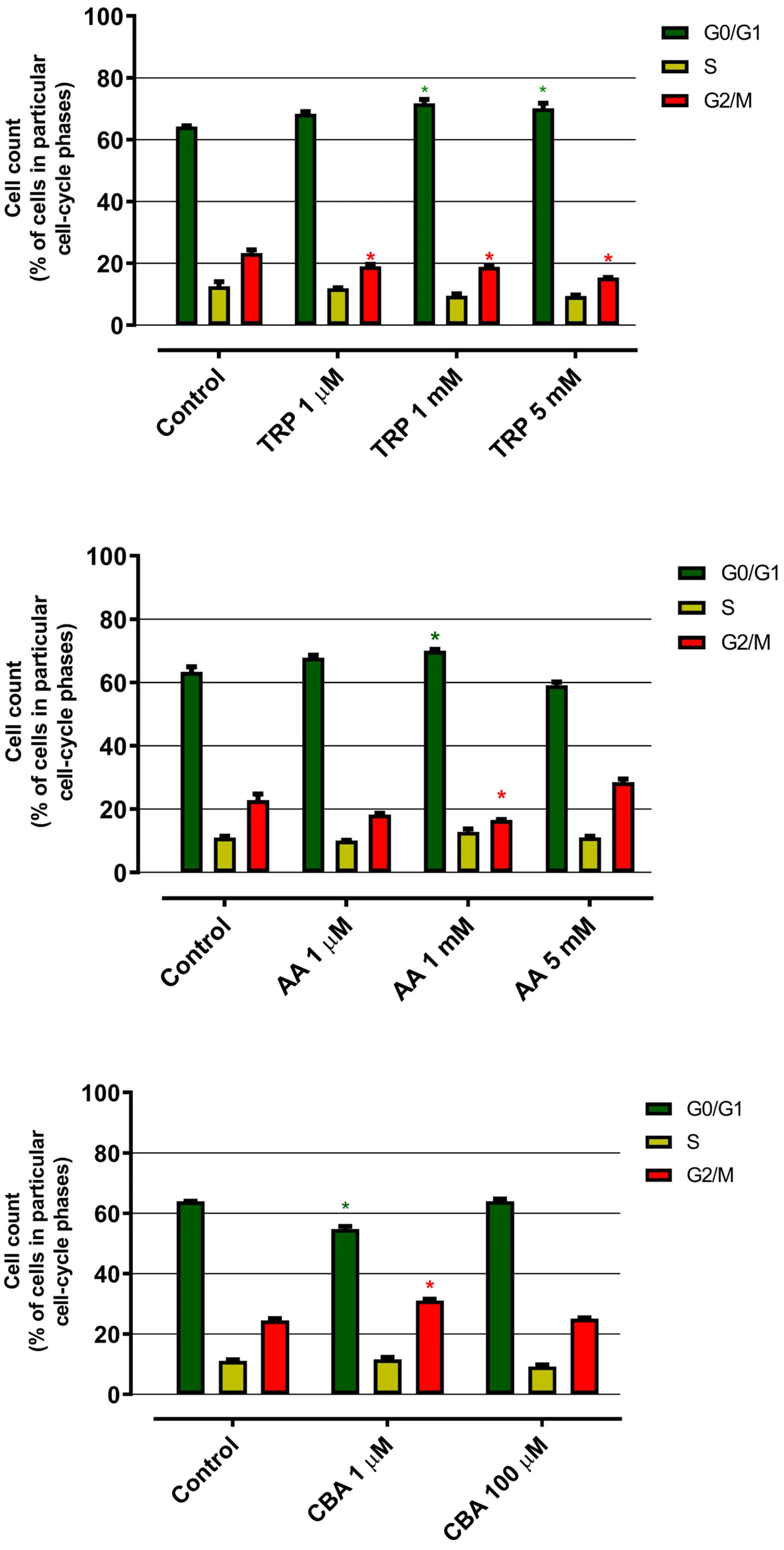
The effect of TRP and its selected metabolites on cell-cycle progression of melanoma A375 cells. A375 cells were exposed to a range of concentrations of TRP, AA (1 µM, 1 mM, 5 mM), and CBA (1 µM, 100 µM) for 24 h. The effect of the tested substances on the cell-cycle progression was determined by flow cytometry after cell staining with propidium iodide. Data are presented as the mean value and standard deviation (SD) of two independent trials. Data are presented as % of control (Control—non-treated, 100%); statistical significance (one-way ANOVA with Dunnett post hoc test, *p* < 0.05 (*)).

Based on the obtained biological activity results (Figure 2) and the well-documented characteristics of the A375 melanoma-cell line, this line was selected for further studies. To evaluate the effect of TRP and its selective metabolites on cell-cycle progression, A375 cells were incubated with the tested compounds for 24 h. The concentrations of the substances for further studies were selected based on the results of the MTT Assay. Flow cytometry revealed the inhibitory effect of TRP, AA, and CBA on melanoma-cell progression (Figure 3). TRP significantly increased the population of G0/G1 cells in comparison to untreated cells by 7.56% (1 mM) and 5.91% (5 mM). Moreover, TRP decreased the cell number in G2/M phase by 4.34% (1 µM), 4.47% (1 mM), and 7.96% (1 mM) in comparison to control (Figure 3). A similar pattern of changes was observed in A375 cells exposed to AA at a concentration of 1 mM, which increased cell accumulation in the G0/G1 phase by 6.73% and simultaneously decreased population of G2/M cells by 6.21%. CBA at the concentration of 1 µM decreased the number of cells in the G0/G1 phase by 9.11% (Figure 3). CBA at a concentration of 1 µM increased cell accumulation in the G2/M phase by 6.57% (Figure 3).

**Figure 4:**
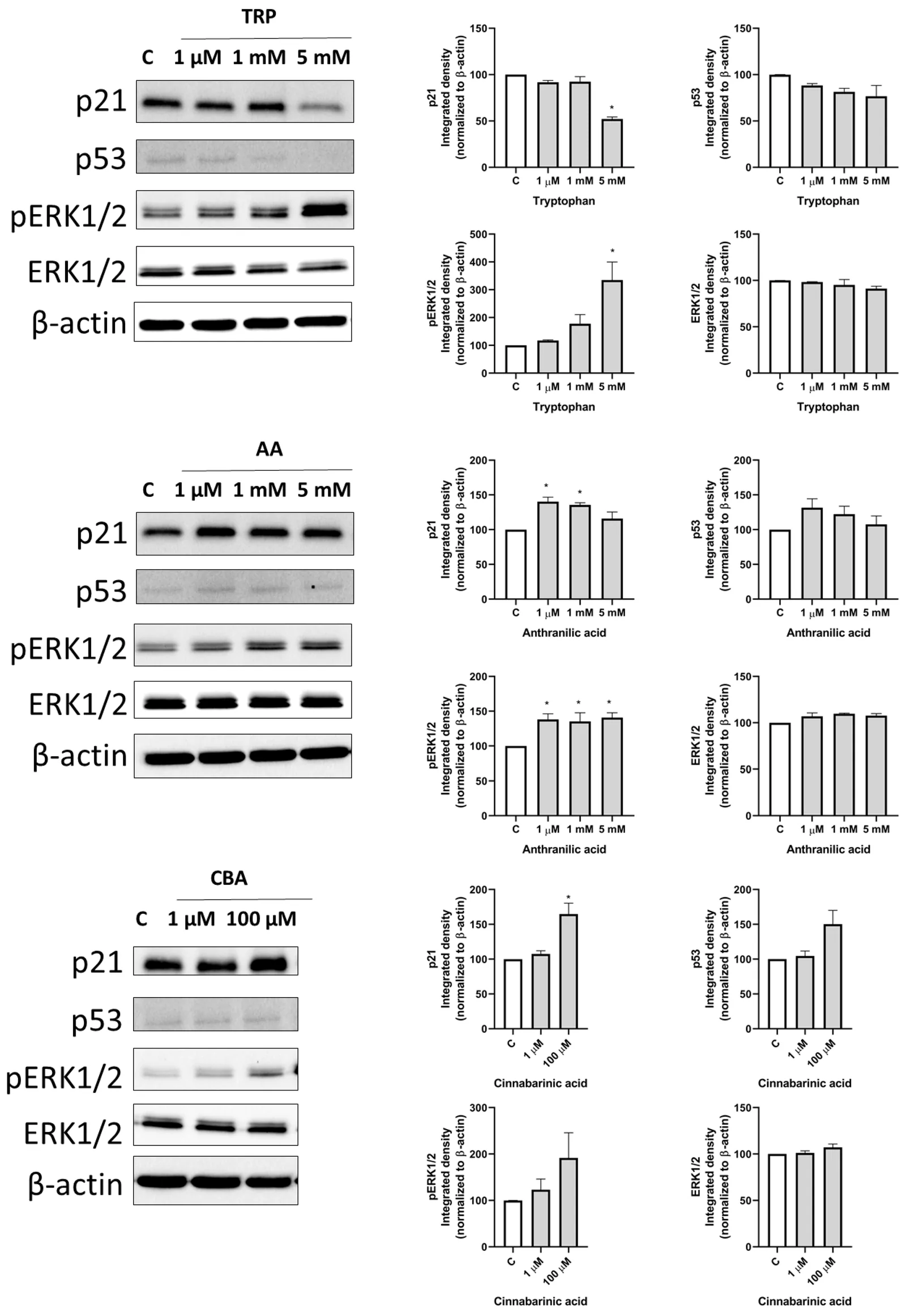
The effect of TRP and its selected metabolites on the protein levels of selected cell-cycle regulators and activation of ERK1/2 kinase in melanoma A375 cells. A375 cells were exposed to a range of concentrations of TRP, AA (1 µM, 1 mM, 5 mM), and CBA (1 µM, 100 µM) for 24 h. The effects of the tested substances on the protein levels of selected cell-cycle regulators (p21, p53) and activation of ERK1/2 kinase were determined using western blotting. Western blots were selected as the most representative of the series of repetitions (*N* = 3, independent biological replicates). The results of densitometric analysis, normalized relative to β-actin, are shown as a relative value of the control (Control—non-treated, 100%). * statistical significance (one-way ANOVA with Tukey post hoc test, *p* < 0.05).

Previous studies revealed that TRP metabolites are involved in the regulation of selected signaling pathways and cell-cycle regulation in melanoma and other cancer cells [1,3,17]. Importantly, Fröhlich et al. revealed that the effectiveness of targeted therapies in melanoma cells is dependent on p21 protein level [32]. Thus, the effect of TRP, AA, and CBA on protein expression of selected cell-cycle regulators and activation of ERK 1/2 kinase was studied in melanoma A375 cells by western blotting (Figure 4). All tested substances affected the protein level of the inhibitor of cyclin-dependent kinases p21, and the effect was dependent on the substance and its concentration. TRP at the highest tested concentration significantly decreased the level of p21 and increased phosphorylation of ERK1/2, whereas AA increased the protein level of p21 and activation of ERK1/2 kinase. CBA at a concentration of 100 µM significantly increased the level of p21. Moreover, we did not observe any effect of TRP, AA, and CBA on the protein expression of p53.

**Figure 5:**
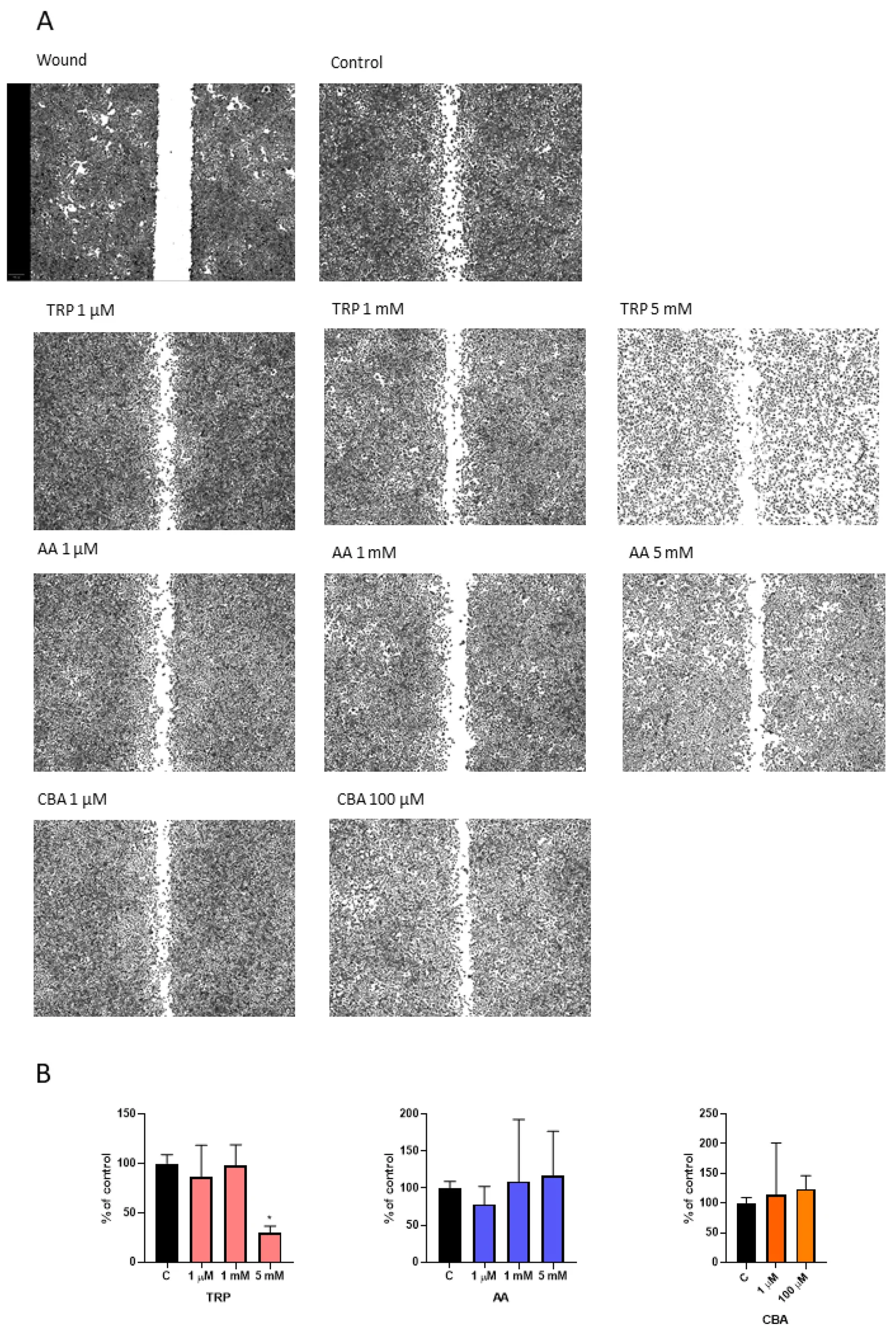
The effect of TRP and its selected metabolites on melanoma A375 migration. Scratched monolayers of A375 cells were exposed to a range of concentrations of TRP (1 µM, 1 mM, 5 mM), AA (1 µM, 1 mM, 5 mM), or CBA (1 µM, 100 µM) for 24 h. (**A**) Micrographs show representative images (magnification ×20). (**B**) Melanoma cells in the wound area were counted. Data are presented as % of control (Control—non-treated, 100%). * statistical significance (one-way ANOVA with Tukey post hoc test, *p* < 0.05). Scale bar = 250 µm.

The effect of TRP and its selected metabolites, AA and CBA, on migration of melanoma A375 cells was determined by the scratch assay (Figure 5). TRP at millimolar concentrations inhibited migration of A375 cells after 24-h exposure; however, we did not observe a significant effect in melanoma cells exposed to AA and CBA.

**Figure 6:**
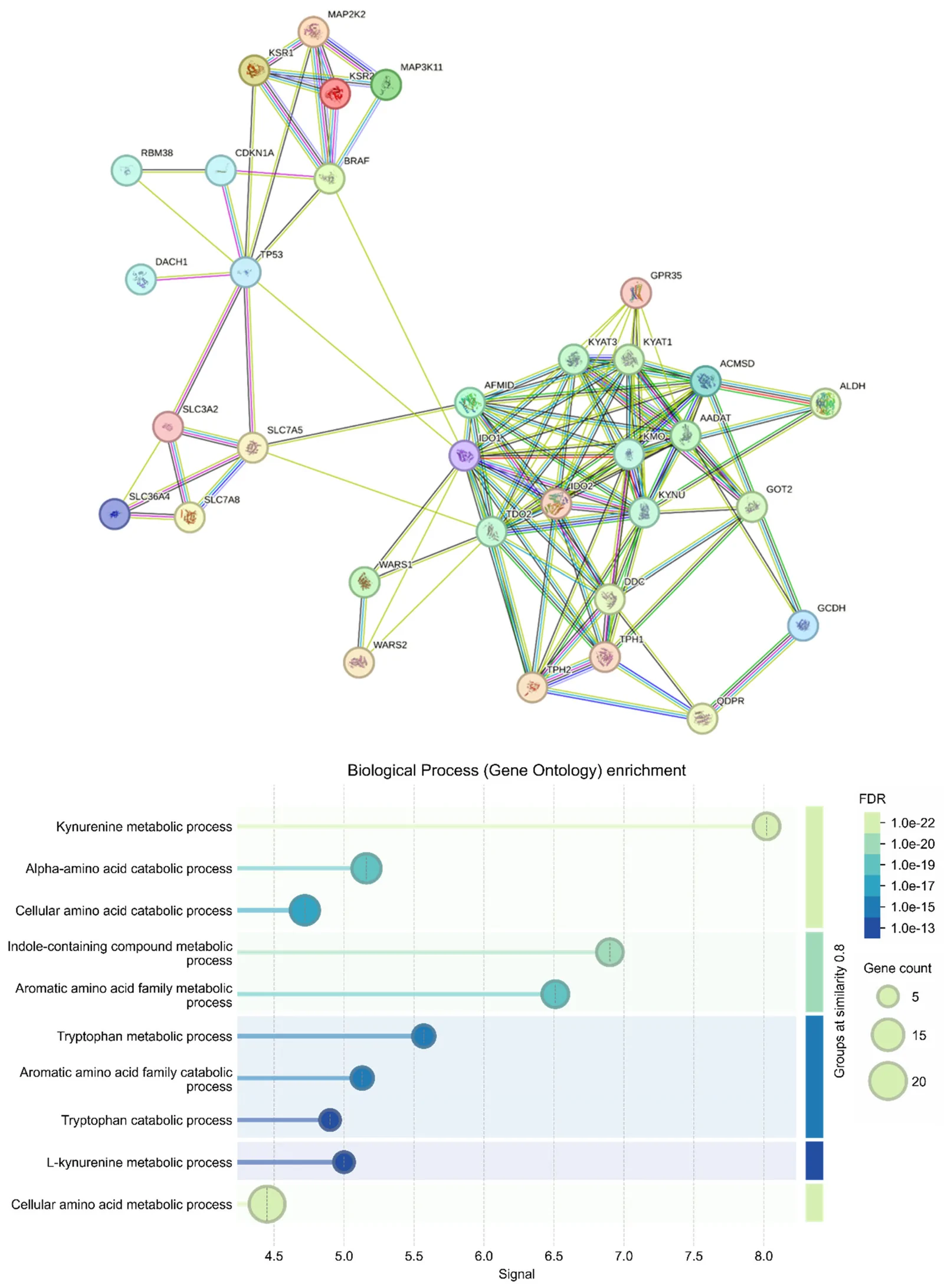
The protein–protein interaction network between *BRAF*, *TRP*, *KYNU*, *KMO*, *CDKN1A*, and *TP53* [34,35]. Edges represent protein–protein associations. The bubble plot summarizes the enrichment analysis results. Bubble size represents the number of genes associated with each term (gene count), while color indicates the statistical significance expressed as the false discovery rate (FDR). Larger bubbles correspond to terms containing more genes, and lighter colors indicate stronger statistical significance (lower FDR values). Terms located further to the right show a higher enrichment score, suggesting a stronger association with the analyzed gene set. Known Interactions: ---- from curated databases, ---- experimentally determined; Predicted Interactions: ---- gene neighborhood, ---- gene fusions, ---- gene co-occurrence; Others: ---- text mining, ---- co-expression, ---- protein homology. Nodes: *AFMID* Kynurenine formamidase; *IDO1* Indoleamine 2,3-dioxygenase 1; *DDC* Aromatic-L-amino-acid decarboxylase; *QDPR* Dihydropteridine reductase; *WARS2* Tryptophan--tRNA ligase; *TPH1* Tryptophan hydroxylase 1; *KSR2* Kinase suppressor of Ras 2; *KYAT3* Kynurenine--oxoglutarate transaminase 3; *GCDH* Glutaryl-CoA dehydrogenase; *BRAF* Serine/threonine-protein kinase B-raf; *SLC7A8* Large neutral amino acids transporter small subunit 2; *ALDH8A12*-aminomuconic semialdehyde dehydrogenase; *GOT2* Aspartate aminotransferase; *CDKN1A* Cyclin-dependent kinase inhibitor 1; *TDO2* Tryptophan 2,3-dioxygenase; *KYNU* Kynureninase; *KMO* Kynurenine 3-monooxygenase; *KSR1* Kinase suppressor of Ras 1; *SLC36A4* Proton-coupled amino acid transporter 4; *SLC3A24F2* cell-surface antigen-heavy chain; *GPR35* G-protein-coupled receptor 35; *SLC7A5* Large neutral amino acids transporter small subunit 1; *ACMSD2*-amino-3-carboxymuconate-6-semialdehyde decarboxylase; *MAP3K11* Mitogen-activated protein kinase kinase kinase 11; *IDO2* Indoleamine 2,3-dioxygenase 2; *TP53* Cellular tumor antigen p53; *AADAT* Kynurenine/alpha-aminoadipate aminotransferase; *TPH2* Tryptophan hydroxylase 2; *KYAT1* Kynurenine--oxoglutarate transaminase 1; *MAP2K2* Dual-specificity mitogen-activated protein kinase kinase 2; *WARS1* Tryptophan--tRNA ligase, cytoplasmic.

Targeted therapies are currently among the most effective treatments for melanoma, including BRAF inhibitors. *BRAF* mutations are detected in approximately 50% of melanomas. Activation of BRAF-dependent signaling pathways may lead to an increase in proliferation and survival of cancer cells [33]. We generated data concerning the functional interaction network between *BRAF* and TRP-associated genes using the STRING database [34,35]. We revealed the functional interactions between *BRAF* and TRP metabolism (i.e., *IDO1*, *IDO2*, *TDO2*, *KYAT1*, *AADAT*, *KYAT3*, *GOT2*, *AFMID*, *KYNU*, *KMO*, *ACMSD*), transporters and receptors (i.e.,: *SLC7A5*, *SLC3A2*, *SLC7A8*, *SLC36A4*, *GPR35*), cell-cycle regulators (i.e.,: *TP53*, *CDKN1A*) and signaling pathways (i.e.,: *KSR1*, *KSR2*, *MAP2K2*, *MAP3K11*) (Figure 6). Enrichment analysis revealed a strong overrepresentation of genes involved in amino acid metabolism. The broad term cellular amino-acid metabolic process indicates a global reprogramming of amino acid metabolism, whereas the highly enriched kynurenine metabolic process suggests a specific involvement of TRP catabolism and the KP. These findings point to substantial metabolic adaptations affecting both general amino acid turnover and specialized pathways linked to energy metabolism, immune regulation, and cellular stress responses. The detailed data concerning the interactions between genes are presented in Supplementary Materials (Table S1).

**Figure 7:**
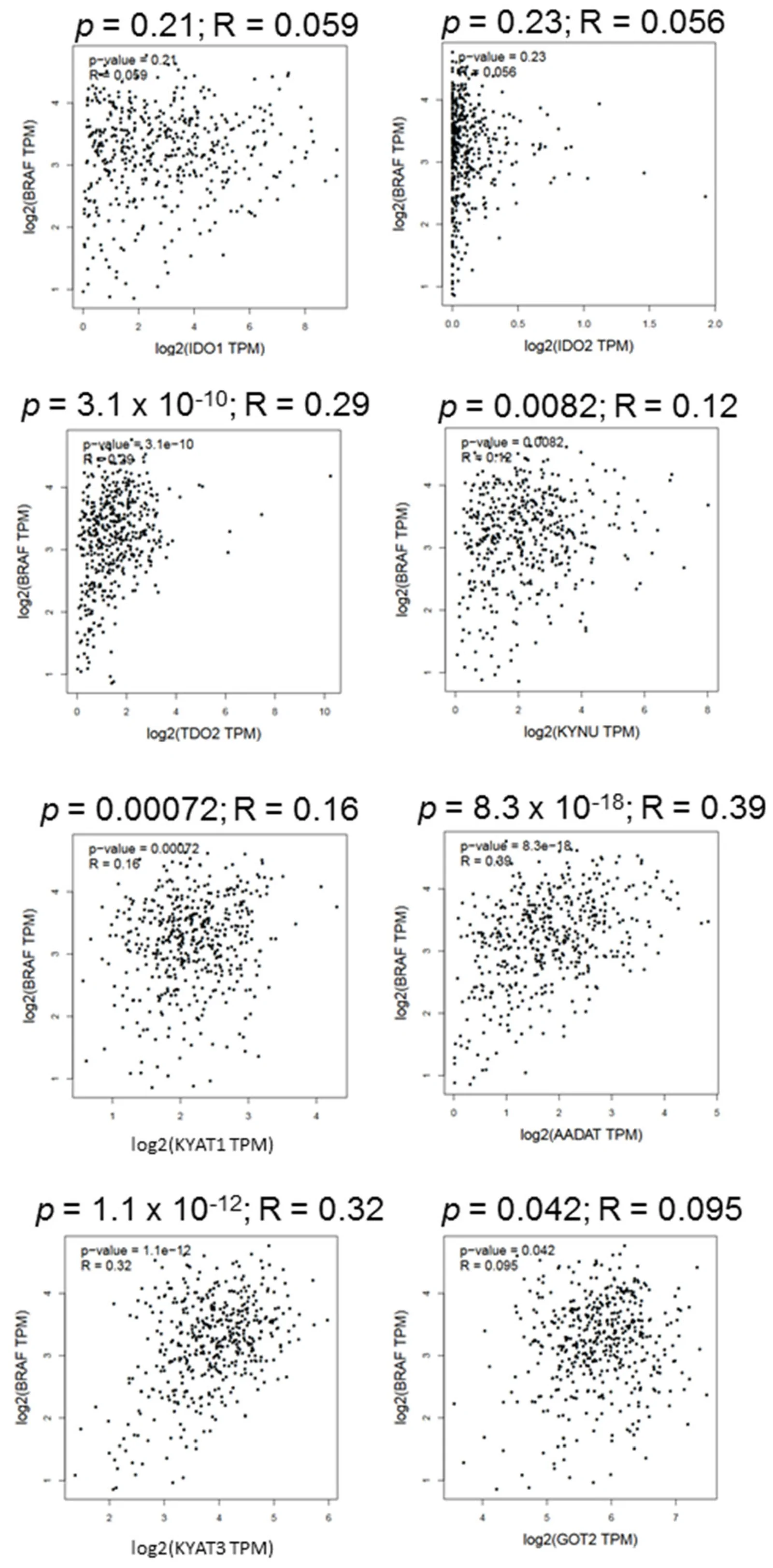
The correlation between the expression of *BRAF* and selected genes coding the enzymes of the KP in skin cutaneous melanoma. Data and graphs were generated by GEPIA2 (the Cancer Genome Atlas (TCGA) data) and statistically analyzed by Spearman correlation (*p* < 0.05) [25,36]. Retrieved expression values are provided in transcripts per million (TPM). *BRAF* B-Raf proto-oncogene, serine/threonine kinase, *IDO1* indoleamine 2,3-dioxygenase 1, *IDO2* indoleamine 2,3-dioxygenase 2, *TDO2* tryptophan 2,3-dioxygenase, *KYNU* kynureninase, *KYAT1* kynurenine aminotransferase 1, *AADAT* aminoadipate aminotransferase, *KYAT3* kynurenine aminotransferase 3, *GOT2* glutamic-oxaloacetic transaminase 2.

Moreover, we decided to investigate a relationship at the genetic level between *BRAF* and selected KP genes in melanoma. The data obtained from GEPIA2 [36] revealed a statistically significant correlation between the expression of *BRAF* and *TDO2*, *KYNU*, and all genes coding kynurenine aminotransferases (KATs) (*KYAT1*, *AADAT*, *KYAT3*, and *GOT2*) (Figure 7). However, the Spearman correlation coefficients (R) indicated a weak association between the investigated genes.

**Figure 8:**
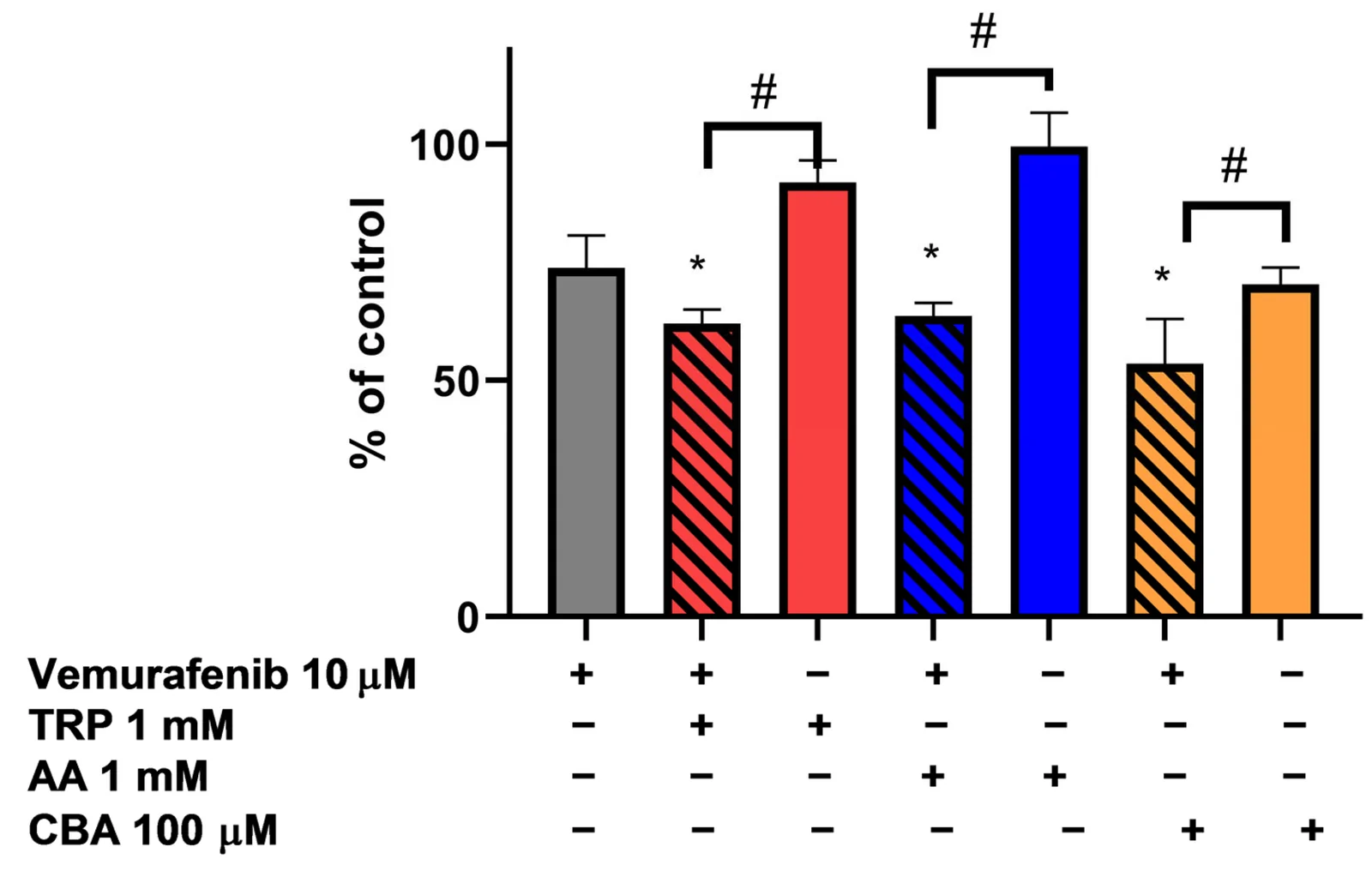
The effect of TRP and its selected metabolites on the biological activity of vemurafenib in melanoma A375 cells. A375 cells were exposed to fresh cell-culture medium (control), TRP (1 mM), AA (1 mM), CBA (100 µM), and vemurafenib (10 µM), or combinations of TRP and its metabolites with vemurafenib for 24 h. The effect of tested substances on A375 proliferation and metabolic activity was assessed by MTT assay. Data are presented as the mean value (*N* = 8, independent biological replicates) and standard deviation (SD). Data are presented as % of control (Control—untreated, 100%). * statistical significance vs. vemurafenib; # statistical significance vs. indicated bars (one-way ANOVA with Tukey post hoc test, *p* < 0.05).

Previous studies revealed that the use of BRAF inhibitors as monotherapy may result in the development of melanoma resistance [21]. Vemurafenib is a selective BRAF V600E inhibitor, significantly improving progression-free and overall survival outcomes in BRAF-mutated melanoma. However, its clinical use is limited by acquired resistance and toxicity [37]. Considering the antiproliferative activity of TRP and its selected metabolites in melanoma cells in vitro (Figure 2), we decided to investigate the effect of the tested substances on the activity of vemurafenib. The concentrations of TRP (1 mM), AA (1 mM), and CBA (100 µM) used in this experiment were selected based on antiproliferative activity in A375 cells (Figure 2). The highest concentrations, which could exert non-specific toxic effects, were excluded. All tested compounds enhanced the inhibitory activity of vemurafenib (10 µM) in melanoma A375 cells (Figure 8). CBA at a concentration of 100 μM demonstrated the highest efficacy, enhancing vemurafenib activity by 20.26% after 24-h incubation. TRP and AA exerted a similar effect, increasing the inhibitory activity of vemurafenib by 11.84% and 10.21%, respectively (Figure 8).

**Figure 9:**
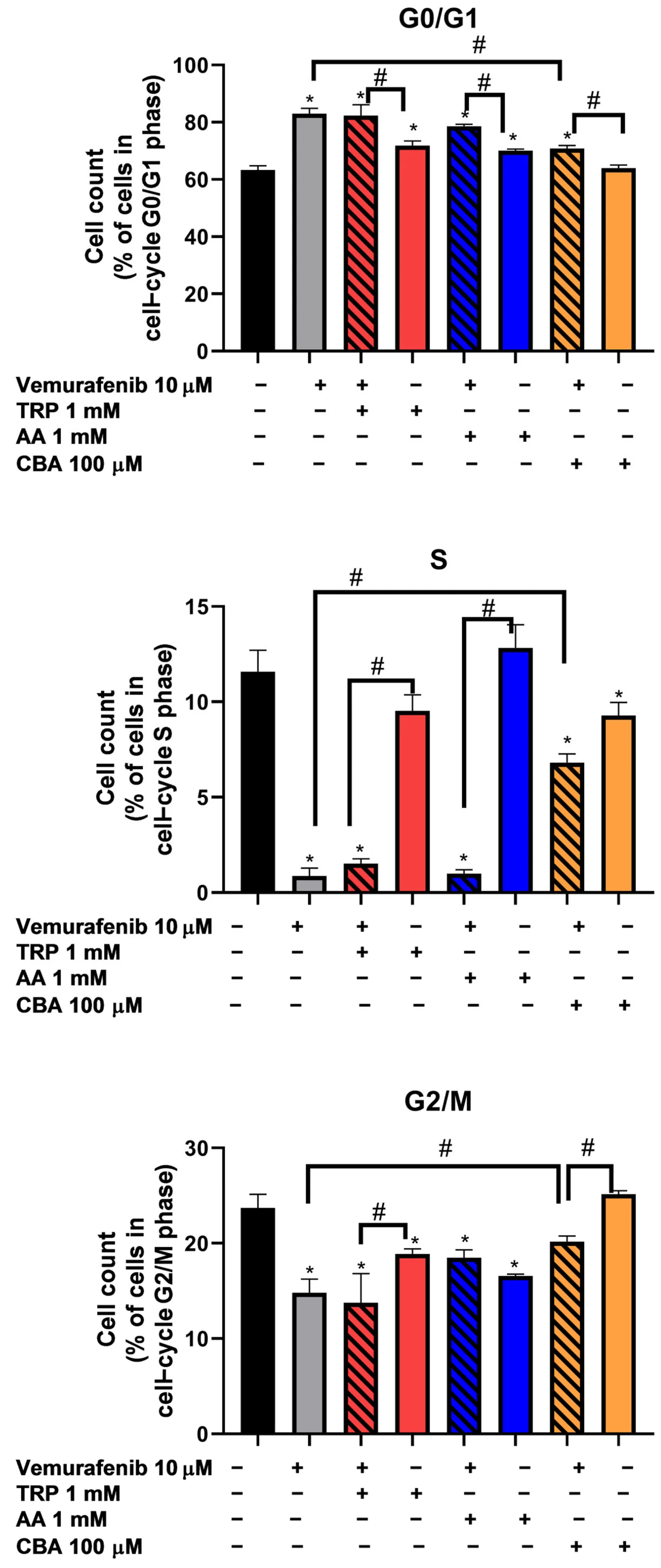
The effect of TRP and its selected metabolites on cell-cycle progression of melanoma A375 cells exposed to vemurafenib. A375 cells were exposed to vemurafenib (10 µM), TRP (1 mM), AA (1 mM), CBA (100 µM), or combinations of TRP and its metabolites with vemurafenib for 24 h. The effect of tested substances on cell-cycle progression was determined by flow cytometry after cell staining with propidium iodide. Data are presented as the mean value and standard deviation (SD) of two independent trials. Data are presented as % of control (Control—non-treated); * statistical significance vs. control; # statistical significance vs. indicated bars (one-way ANOVA with Tukey post hoc test, *p* < 0.05).

Cell-cycle analysis allowed for the evaluation of functional interactions between TRP, its selected metabolites, and vemurafenib in melanoma A375 cells. Vemurafenib (10 µM) inhibited the progression of the cell cycle in A375 cultures, arresting cells in G0/G1 phase (Figure 9). A significant decrease in cell numbers in both S and G2/M phases in A375 cells exposed to vemurafenib was observed. TRP and AA at a concentration of 1 mM did not affect the inhibitory effect of vemurafenib on cell-cycle progression in A375 cells. However, co-incubation with CBA (100 µM) and vemurafenib decreased the population of G0/G1 cells and increased the cell number in both S and G2/M phases in comparison to vemurafenib (Figure 9).

**Figure 10:**
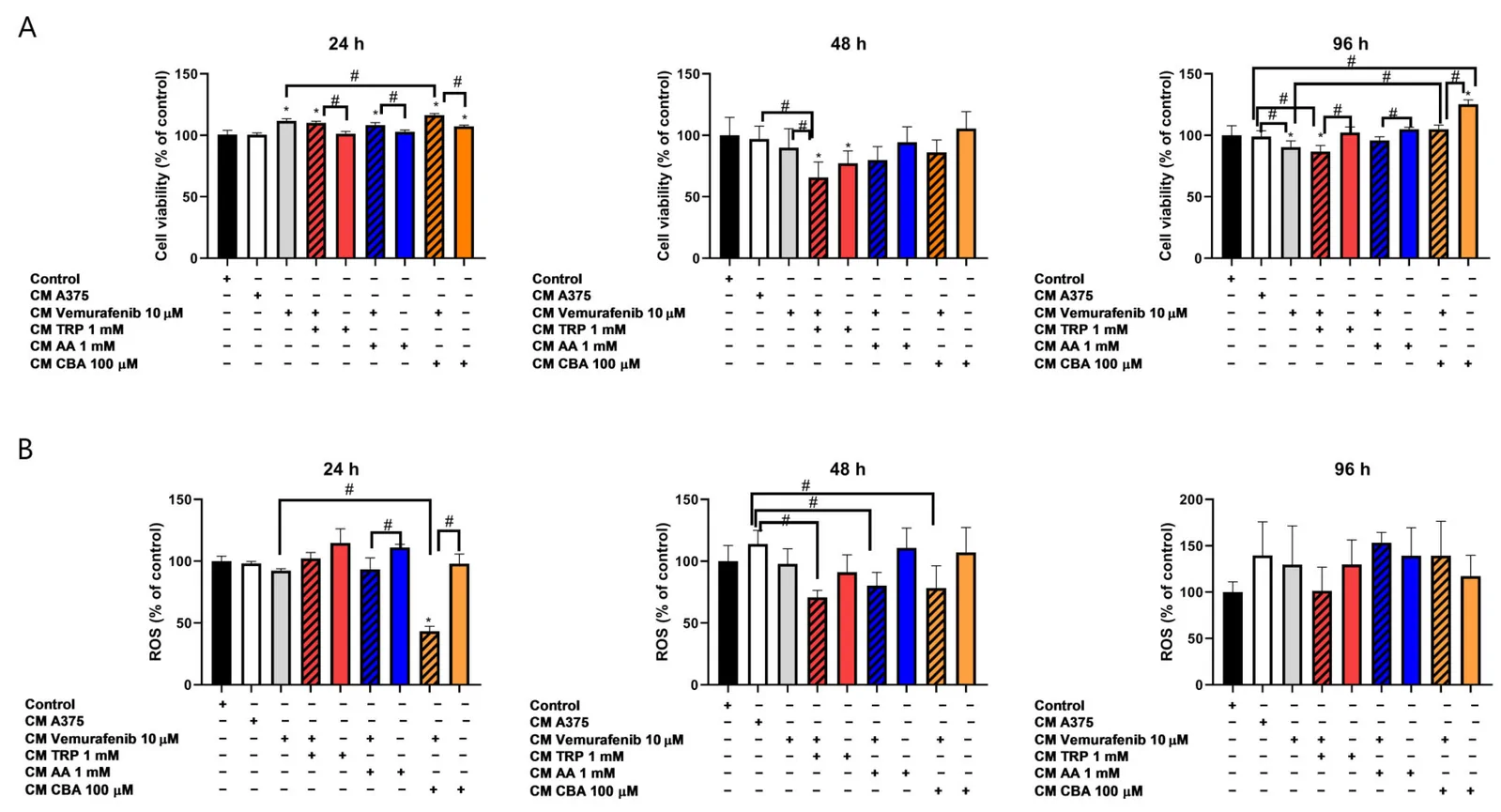

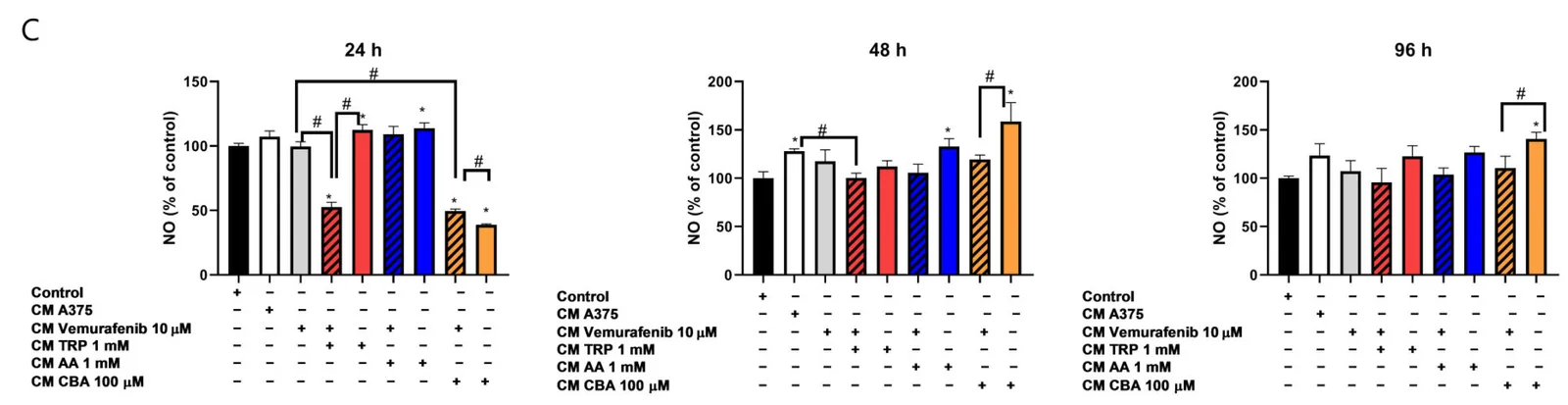
The effect of conditioned medium (CM) from A375 cells exposed to vemurafenib, TRP, and its selected metabolites on cell viability (**A**), ROS production (**B**), and NO production (**C**) by macrophages. PMA-induced THP-1 cells, representing macrophages, were exposed to A375 CM (50% of end-point culture medium) for 24, 48, or 96 h. The effect of CM on cell viability was assessed using the MTT assay (N ≥ 5). The effect of CM on ROS production was determined by DCF-DA (N ≥ 3). The effect of CM on NO production was determined by DAF-FM DA (*N* = 3). Data are presented as the mean value (biological replicates) and standard deviation (SD). Data are presented as % of control (Control—without CM); * statistical significance vs. control; # statistical significance vs. indicated bars (one-way ANOVA with Tukey post hoc test, *p* < 0.05).

Previous studies have demonstrated a significant role of the immune system in melanoma progression. Tumor-associated macrophages (TAMs), the primary immune component of the tumor microenvironment, are considered critical mediators of cancer development, progression, and metastasis [38]. Therefore, we investigated the effects of TRP and its selected metabolites in a functional in vitro macrophage-melanoma-cell model. PMA-induced THP-1 cells were used as an in vitro model of macrophages. Melanoma A375 cells were exposed to vemurafenib (10 µM), TRP (1 mM), AA (1 mM), CBA (100 µM), or combinations of TRP and its metabolites with vemurafenib for 24 h. Cell culture supernatants (CM—conditioned medium) were added to macrophages at a final volume of 50%. CM itself did not affect the viability and metabolic activity of macrophages (Figure 10A). The statistically significant increase in cell viability of macrophages after 24-h exposure to CM-Vemurafenib should be considered as the result of increased metabolic activity. PMA-induced THP-1 cells differentiated into macrophages are characterized by cell-cycle arrest. Similarly, exposure to CM-Vemurafenib/TRP and CM-Vemurafenib for 24 h led to increased metabolic activity of macrophages, but no changes in cell viability were observed after exposure to CM-TRP and CM-AA (Figure 10A). These results indicate changes in metabolic activity in response to changes in the A375 cell secretome following exposure to vemurafenib, but not to TRP and AA alone. In contrast, CM-Vemurafenib/CBA increased cell viability and metabolic activity of macrophages after 24 h, which was the result of the interaction of both CM-Vemurafenib and CM-CBA, as the results were statistically significant in comparison to CM-Vemurafenib and CM-CBA (Figure 10A). Importantly, these observations indicated that the effects are mediated by changes in the secretome of A375 cells exposed to tested compounds, rather than the direct activity of the substances on macrophages. TRP, AA, and CBA did not decrease macrophage viability within the tested concentration range (Figure S1).

However, the biological activity of CM in macrophages was time-dependent. CM from A375 cells exposed to TRP at a concentration of 1 mM decreased the cell viability of macrophages after 48 h (Figure 10A). A similar effect was observed in CM from A375 cells exposed to TRP (1 mM) and vemurafenib (10 µM). After 96-h incubation with CM, the viability of macrophages was decreased in cells exposed to CM vemurafenib (10 µM) and CM vemurafenib (10 µM) and TRP (1 mM) in comparison to CM without tested substances (Figure 10A). Interestingly, a decrease in macrophage viability was observed following incubation with CM from the combination of vemurafenib and TRP or its metabolites, compared to CM containing the individual substances (TRP, AA, CBA) (Figure 10A).

Thus, the observed increase in macrophages treated with CBA-CM after 96 h was a result of enhanced cellular metabolism and should not be attributed to an increase in proliferation (Figure 10A).

To investigate the activity of the macrophages treated with the tumor CM, we analyzed the production of ROS and NO. CM from A375 without any additional compounds (CM A375) and CM-Vemurafenib did not affect ROS production in macrophages (Figure 10B). The decrease in ROS production was observed in macrophages exposed to CM-Vemurafenib/AA and CM-Vemurafenib/CBA after 24-h exposure in comparison to CM-AA and CM-CBA, respectively (Figure 10B). Moreover, CM-Vemurafenib/CBA significantly inhibited ROS production in macrophages in comparison to control and CM-Vemurafenib after 24 h. Importantly, TRP, AA, and CBA did not affect ROS production in macrophages (Figure S2). However, the effect was time-dependent. CM from A375 cells exposed to a combination of vemurafenib and TRP, AA, or CBA decreased ROS production in macrophages after 48 h (Figure 10B). However, no significant effect was observed after 96 h (Figure 10B).

CM-A375 showed a trend toward enhancing NO production in macrophages, with statistically significant effects limited to the 48-h incubation (Figure 10C). 24-h exposure to CM-Vemurafenib/TRP and CM-Vemurafenib/CBA inhibited NO production in macrophages in comparison to CM-Vemurafenib. Importantly, although TRP, AA, and CBA did not affect NO production in macrophages (Figure S3), CM-TRP and CM-AA increased NO production in comparison to control after 24 h (Figure 10C). However, the effect was time-dependent. CM-CBA inhibited NO production after 24 h, whereas the opposite effect was observed after 48 and 96 h (Figure 10C). A significant increase was also observed after 48-h exposure to CM-AA (Figure 10C).

## 3. Discussion

The majority of studies focused on the immunomodulatory effects of TRP and its metabolites in cancers. To our knowledge, this is the first study to confirm direct interactions between TRP, AA, CBA, and melanoma A375, G361, and SK-MEL-3 cells in vitro. A375 and G361 cell lines represent primary melanoma, whereas the SK-MEL-3 cell line was derived from metastatic melanoma in a lymph node. All melanoma-cell lines used in this study bear mutation in the *BRAF* gene (V600E), but only SK-MEL-3 cells do not possess a functional *TP53* gene. A375 cells are characterized by high invasive potential and rapid proliferation, in comparison to G361 cells, which exhibit moderate migratory and invasive potential in vitro [39]. Moreover, taking into consideration previous studies, well-documented characteristics, and cellular activity, the A375 cell line could be considered the most common in vitro model of primary melanoma. Therefore, A375 cells were selected for further experiments.

The results suggest that TRP and its metabolites may play a crucial role in melanoma initiation, progression, and metastasis. Previous studies revealed that some TRP metabolites, including KYN, KYNA, and FICZ, exerted antiproliferative activity against melanoma cells in vitro [1]. To date, selected TRP metabolites, AA and CBA, have not been investigated as potential modulators of melanoma-cell activity. Furthermore, AA is a product of the conversion of KYN—a TRP metabolite with proven effects on melanoma cells in vitro. KYNU mediates the enzymatic conversion of KYN to AA, while also participating in the synthesis of 3-hydroxyanthranilic acid—the direct precursor to CBA [40]. Notably, KYNU expression correlates with BRAF expression in skin-cutaneous melanoma (Figure 7).

In this study, we revealed the inhibitory effect of TRP on the proliferation and metabolic activity of melanoma A375, G361, and SK-MEL-3 cells (Figure 2). The effectiveness of TRP activity increased over time, reaching over 98% inhibition after 96 h of exposure in all melanoma-cell lines. In this study, we used the MTT assay, which appears to be more sensitive in determining the biological activity of TRP metabolites. A previous study demonstrated that TRP metabolites, KYN and KYNA, had a more potent effect on melanoma-cell metabolism than on DNA synthesis [3]. The MTT assay results should be considered as a sum of various biological activities, including cell proliferation, viability, metabolic activity, and cell growth phase [31]. Cell cycle analysis revealed that TRP induced G0/G1 cell cycle arrest in melanoma A375 cells (Figure 3). The accumulation of cells in the G0/G1 phase was accompanied by a simultaneous decrease in the cell number in the G2/M phase after 24 h of incubation with TRP. Therefore, activation of ERK 1/2 kinase in A375 cells exposed to TRP at a concentration of 5 mM should be interpreted as a cellular response to stress signals, taking into consideration the inhibitory activity of TRP in proliferation and migration of melanoma A375 cells (Figure 3 and Figure 4).

Moreover, for the first time, the antiproliferative activity of AA in melanoma A375, G361, and SK-MEL-3 cells was determined (Figure 2). However, the inhibitory potential of AA was markedly lower than that observed for TRP and CBA. Analysis of cell-cycle progression revealed cell cycle arrest in the G0/G1 phase in cultures of A375 cells exposed to 1 mM AA for 24 h, which corresponds to the data obtained following TRP treatment (Figure 3). The molecular mechanism of antiproliferative activity of AA may result from the upregulation of the cyclin-dependent kinase inhibitor p21 (Figure 4), which is considered a predictive biomarker in melanoma patients [32]. The p21 protein inhibits cell-cycle progression via direct interaction with CDK4/6-cyclin D and CDK2-cyclin E complexes. Importantly, previous studies reported that pro-longed activation of the ERK pathway may induce enhanced expression of p21 [41], which was also observed in A375 cells exposed to AA (Figure 4). However, the direct effect of AA on cancer cells remains incompletely understood. AA might be considered a biomarker of advanced gastric cancer, because its concentration was significantly higher in the peritoneal lavage washings in patients diagnosed with metastatic gastric cancer than in patients with early-stage disease [42]. The concentration of this metabolite was also increased in a mouse model of early-stage lung adenocarcinoma [43]. The biological activity of AA derivatives is very broad, including interactions with aldo-keto reductase enzymes, and the hedge-hog and MAPK5 signaling pathways [44]. Several studies revealed anticancer activity of AA derivatives affecting proliferation, cell death, and cell-signaling pathways in cancer cells [45,46,47].

Our results, for the first time, focused on the biological effect of CBA on melanoma cells. CBA, formed by spontaneous autoxidation of 3-hydroxyanthranilic acid, is considered an AhR ligand [48]. Although CBA is not as effective of an activator of the basic AhR pathway leading to the upregulation of CYP1A1 as other TRP metabolites, such as KYN and KYNA, it significantly increased the level of IL-22 in CD4+ cells [49]. Interestingly, a high expression profile of IL-22 in melanoma tumors was correlated with immunotherapy responses [50]. Previous studies revealed that the IL-22 receptor was also involved in the antiangiogenic activity of sMDA-7/IL-24 [51]. The antiproliferative activity of CBA in melanoma A375 cells (Figure 2) might result from the inhibition of mitochondrial respiration. The MTT assay indirectly assesses the effect of the tested compound on proliferation by measuring cellular metabolic activity. Previous studies reported that CBA inhibited state III mitochondrial respiration more potently than 3-hydroxyanthranilic acid [52,53]. Moreover, it was suggested that the effect of CBA on cell survival might be dependent on cell type and the time of exposure [54,55,56]. Cell-cycle analysis and protein expression of selected cell-cycle regulators and components of the ERK 1/2 signaling pathway revealed a modulatory role of CBA in melanoma A375 cells (Figure 3 and Figure 4). CBA at 1 µM induced cell-cycle arrest in G2/M phase in A375 cultures, with a concomitant decrease in the G0/G1 population (Figure 3). The changes at the cellular level were accompanied by molecular alterations in p21 protein expression levels (Figure 4).

The upregulation of BRAF-dependent signaling cascades is known to promote the proliferation and survival of melanoma cells. Thus, therapies targeting BRAF have emerged as the most effective treatment for melanoma, which exhibits *BRAF* mutations in nearly 50% of patients [32]. We performed the bioinformatics analysis to investigate whether TRP and its metabolites may interact with BRAF inhibitors. Interestingly, we found a wide network of BRAF interactions not only between the KP genes (*IDO1*, *IDO2*, *TDO2*, *KYNU*, *KMO*, *KYAT1*, *AADAT*, *KYAT3*, *GOT2*, *ACMSD*), but also transporters (*SLC3A2*, *SLC7A5*, *SLC7A8*, *SLC36A4*), receptors (*GPR35*), cell-cycle regulators (*TP53*, *CDKN1A*), and signaling pathways (*MAP2K2*, *MAP3K11*, *KSR1*, *KSR2*) (Figure 6). The STRING analysis identified associations between BRAF and several proteins involved in MAPK signaling, providing a potential mechanistic context for the observed effects of vemurafenib. Importantly, the bioinformatic analysis revealed the interaction between BRAF, the KP, and cell-cycle regulators, which may be relevant to melanoma biology (Figure 6 and Figure 7). The level of p21, a cyclin-dependent kinase inhibitor 1A coded by *CDKN1A*, is considered a predictive biomarker in melanoma patients [32]. Moreover, it was suggested that activation of p53 might increase the efficacy of treatment with BRAF inhibitors by p21 upregulation [32]. TRP metabolites, including KYNA, KYN, and 8-hydroxyquinaldic acid, increased the level of p21 in various cancer cells in vitro [57,58,59]. Similar activity of AA and CBA (100 µM) was observed in A375 cells (Figure 4). Importantly, our research revealed that TRP, AA, and CBA increased the phosphorylation of ERK1/2 kinase in A375 cells (Figure 4). However, we did not observe any stimulation of cancer-cell proliferation. Therefore, activation of ERK kinase may be the result of cellular stress. Similarly, previous studies reported the activation of ERK1/2 mediated by the calcium-sensing receptor in HEK-293 cells exposed to TRP [60]. It cannot be excluded that the tested compounds might also interact with other MAP kinases. The inhibitory effect on ERK1/2 and p38 activity of KYNA, another TRP metabolite, was previously observed in colon-cancer cells [17].

To verify whether TRP and its derivatives might interact with the activity of BRAF inhibitors in melanoma, A375 cells were exposed to vemurafenib, TRP, AA, and CBA (Figure 8). The increased antiproliferative effect of vemurafenib (10 µM) was observed after co-exposure with TRP (1 mM), AA (1 mM), and CBA (100 µM) (Figure 8). Interestingly, antiproliferative activity of selected concentrations of AA and CBA was correlated with increased p21 level in A375 cells (Figure 4), which confirmed the hypothesis of Fröhlich et al. [32]. However, despite the enhanced activity of vemurafenib in A375 cells exposed to TRP, this amino acid did not upregulate p21 (Figure 4), which may suggest a different molecular mechanism. Cell cycle analysis revealed that co-incubation with vemurafenib and TRP or AA did not modify the inhibitory effect of vemurafenib, which induced cell cycle arrest in the G0/G1 phase in cultures of A375 cells (Figure 9). Therefore, it should be suggested that the inhibitory potential of TRP, AA, and CBA observed in co-incubation with vemurafenib (Figure 8) might result from an additional metabolic mechanism.

It should be underlined that cancer cells exist within a complex network of interactions with healthy and immune cells, creating a privileged microenvironment. A decrease in essential nutrients, including glucose, glutamine, arginine, and TRP, in the tumor microenvironment is another hallmark of cancer [61]. Thus, it cannot be excluded that TRP and its metabolites may be potential metabolic signaling biomarkers between cancer cells and the tumor microenvironment. TAMs, the primary immune component of the tumor microenvironment, are considered critical mediators of cancer development, progression, and metastasis [38]. Previous studies indicated that increased levels of TAMs correlate with poor clinical outcomes and cancer progression. TAMs frequently undergo phenotypic polarization in immunosuppressive cells, thereby stimulating tumor growth, supporting the maintenance of cancer stem cells, and driving key oncogenic processes such as angiogenesis and metastasis (reviewed in [38,62,63]). Macrophage plasticity enables differentiation from a pro-inflammatory phenotype (M1) to immunosuppressive cells (M2). M1 macrophages are characterized by the production of pro-inflammatory mediators, including interleukin (IL)-12, IL-1, IL-6, tumor necrosis factor (TNF)-α, ROS, and NO, alongside an upregulation of MHC class II molecules [64]. Conversely, exposure to IL-4, IL-10, and IL-13 leads to phenotype polarization into M2 macrophages, key effectors in humoral immune responses, and physiological processes such as tissue repair and remodeling [63,65]. Although TAMs predominantly exhibit M2 characteristics, recent studies have confirmed that the local tumor microenvironment consists of heterogeneous populations that share characteristics of both M1 and M2 populations [66]. Moreover, previous studies re-ported that melanoma exosomes induced mixed M1 and M2 population and upregulated biomarkers characteristic of both phenotypes, including TNF-α, IL-10, CCL22, IL-12B, IL-1β, IL-6, and iNOS [67]. In this study, the effect of CM-A375 on cell viability, ROS, and NO production by macrophages was revealed (Figure 10). CM-A375 did not affect the viability of macrophages after 24, 48, and 96-h incubation. Interestingly, the increase in cell viability of macrophages exposed to CM-Vemurafenib, CM-Vemurafenib/TRP, CM-Vemurafenib/AA, CM-Vemurafenib/CBA, and CM-CBA should be interpreted as an increase in metabolic activity rather than cellular proliferation (Figure 10A). Following PMA activation, THP-1 cells undergo differentiation and consequently lose their capacity to proliferate. Notably, CM-CBA treatment induced an increase in macrophage viability compared to control cells after 24- and 96-h incubation, whereas CM-TRP and CM-AA exerted no such effects. The results presented in this study indicate a distinct mechanism of biological activity for CBA compared to TRP and AA. This may be due to the fact that CBA is one of the end-products of the kynurenine pathway, while TRP and AA remain susceptible to further enzymatic transformations. Furthermore, the impact of CM on the viability of macrophages was also time-dependent. CM-vemurafenib decreased the viability of macrophages in comparison to control and CM-A375 after 96-h incubation (Figure 10A). Interestingly, the viability of macrophages was decreased after exposure to CM-vemurafenib/TRP and CM-vemurafenib/AA in comparison to CM-TRP and CM-AA, respectively (Figure 10A). Despite belonging to the group of TRP metabolites, CM-CBA exerted the opposite effect on the viability of macrophages. Importantly, the results suggest that TRP and its metabolites modified the profile of components secreted by melanoma A375 cells, rather than exerting direct effects on macrophages (Supplement Figures S1–S3). Several studies focused on the immunomodulatory role of TRP and its metabolites. IDO1, one of the rate-limiting enzymes of TRP metabolism, plays an important role in the immune suppression [68]. Overexpression of IDO1 is observed in various cancers, including melanoma [69], and its upregulation and decreased TRP/KYN ratio resulted in significantly lower survival of melanoma patients [70,71]. However, IDO1 inhibitors did not bring a breakthrough solution in the clinical trials involving cancer patients [72,73]. Notably, TRP belongs to the group of amino acids, including cysteine, leucine, and arginine, which play a crucial role in immune response to melanoma by induction of ferroptosis, inhibition of transporters, or enhanced lipid peroxidation in immune cells [74,75,76]. TRP may affect mRNA translation in melanoma cells through ribosomal frameshifting, leading to presentation of aberrant trans-frame peptides to immune cells [77]. On the contrary, TRP modifies the immune profile in the tumor microenvironment by upregulation of IDO1 and TDO in tumor-infiltrating lymphocytes and melanoma cells. Decreased level of TRP leads to activation of immunosuppressive Tregs and starvation of cytotoxic T cells [78,79]. Melanoma immune escape is also regulated by KYN-dependent AhR activation [80].

To determine the potential metabolic signaling between melanoma cells and macrophages on ROS and NO production, PMA-induced THP-1 cells were exposed to CM from A375 cells treated with tested compounds (Figure 10B,C). The results of ROS production point to a distinct effect of CM-CBA. Macrophages treated with CM-Vemurafenib/CBA for 24 h produced significantly less ROS compared to the control, as well as compared to CM-Vemurafenib and CM-CBA alone. Regarding the effects of CM with the tested substances on ROS production, a time-dependent variation in activity was observed. The CM-vemurafenib/TRP, CM-vemurafenib/AA, and CM-vemurafenib/CBA decreased ROS production in macrophages in comparison to CM-A375 after 48 h (Figure 10B). ROS have a dual role in cancer, exerting either pro- or anti-tumorigenic effects. The imbalance between ROS production and detoxification mechanisms leads to oxidative stress. Melanoma cells adapt to environmental conditions by modifying their bioenergetic metabolism. However, previous studies suggested that ROS produced in mitochondria might be considered signal mediators and affect proliferation, differentiation, cell survival, and cell death [81,82,83].

NO plays a multifaceted role in melanoma progression, impacting key biological processes in melanoma cells, including melanogenesis, angiogenesis, and apoptosis, but also affecting the immune response. A significant inhibition of NO production was observed in macrophages exposed for 24 h to CM-Vemurafenib/TRP, CM-Vemurafenib/CBA, and CM-CBA (Figure 10C). However, the biological activity of CM was highly dependent on the incubation time. CM-A375, CM-AA, and CM-CBA increased NO production in macrophages in comparison to the control after 48-h incubation (Figure 10C). Interestingly, CM-CBA significantly decreased NO production by macrophages after 24 h, whereas 48-h and 96-h incubation resulted in increased NO production. Although elevated NO levels are generally considered a marker of poor clinical outcomes, its regulatory effects are complex; depending on the local concentration, NO may also exert anti-proliferative effects on melanoma cells [84,85]. The level of NO in the tumor microenvironment is the result of endogenous production by tumor cells and cells of the immune system. In BRAF-mutated A375 models, where baseline NO production is low, NO predominantly diffuses from the surrounding environment, including macrophages and other infiltrating immune cells. The clinical significance of this molecule is further complicated by its dose-dependent effects, as elevated concentrations trigger cytotoxicity while lower levels enhance tumor proliferation [85,86]. He et al. revealed that generation of NO led to apoptosis and autophagy in A375-S2 cells [87]. NO may directly and indirectly affect melanoma growth and metastasis. Previous studies revealed the interaction between NO and VEGF expression. NS1, a considered NOS inhibitor, led to G2/M cell-cycle arrest and decreased VEGF expression in melanoma A375 cells, modulating the ERK and PI3K/Akt signaling pathways [88].

Considering the non-selectivity of some of the KP enzymes and their involvement in other biological reactions, the indicated correlation needs further studies to verify whether the KP activation is one of the defense and regulatory mechanisms in melanoma. KP metabolites may act at the transcriptomic and metabolic levels in tumor cells and their environment. These findings suggest that the complex metabolic and transcriptomic balance among TRP, its metabolites, and KP genes in cancer cells, the tumor microenvironment, and the immune system, rather than individual genes or metabolites alone, may be critical for understanding the role of this amino acid in cancer development, progression, and metastasis. However, advanced in vivo and clinical studies are necessary to verify this hypothesis. The presented results should be considered a preliminary study indicating a research direction for future investigations. The study was conducted on melanoma A375 cells harboring mutations in *BRAF* (c.1799 T > A) and *CDKN2A* (c.181G > T) genes [89]. *CDKN2A* is considered the most frequently inactivated tumor suppressor gene in melanoma; however, approximately 25% of *BRAF* mutations in melanoma have been identified in a different position of the gene sequence [22,90,91]. Therefore, it is difficult to clearly indicate the molecular mechanism underlying the inhibitory action of TRP and its metabolites on melanoma cells at this stage of the research. Previous studies confirmed the presence of selected genes encoding KP enzymes and the production of KYNA and AA in A375 melanoma cells, indicating the activity of the KP in these cells [92,93]. Therefore, it cannot be excluded that TRP and metabolites exert their effects through complex pathways. The exogenous administration of the KP products or substrates may disrupt the kinetics of enzymatic reactions, leading to significant alterations in cellular metabolic homeostasis. Notably, in this study, antiproliferative activities of TRP, AA, and CBA were observed at higher concentrations. Previous studies revealed their concentrations in human plasma, which varied between 54.40 µM and 66.8 µM for TRP, 12.1 nM and 18.35 nM for AA, and 250 pM and 11,750 pM for CBA [94,95,96,97,98]. Only TRP at a concentration of 1 µM (below the physiological serum concentration) exhibited an inhibitory effect on the proliferation of A375 melanoma cells after 96 h of incubation (Figure 2). However, we did not observe a significant effect of TRP on the cell cycle, migration, or protein expression in melanoma cells within the concentration range corresponding to physiological serum TRP levels (Figure 3, Figure 4 and Figure 5). Despite limited data regarding skin concentrations of the tested metabolites, achieving sufficiently high serum levels appears to be a limiting factor for the potential use of selected TRP metabolites in anticancer therapy. However, topical application directly to the skin may enable high local concentrations and significantly reduce nonspecific effects on normal skin cells.

Importantly, the inhibitory effect of the tested substances on melanoma A375 cells observed at the cellular level may result from a complex network of dependencies at the transcriptomic and metabolic levels, and not only from simple interactions with a single protein. Despite the advantages of using a single cell line to assess the biological activity in a stable genetic environment, such an approach remains a limitation of the study and lacks systemic complexity. Thus, more complex in vitro and in vivo models are required to fully understand the toxicological profile and immunomodulatory effects of TRP and its metabolites.

## 4. Materials and Methods

### 4.1. Drugs

L-TRP, AA, and CBA were obtained from Merck (St. Louis, MO, USA), whereas vemurafenib was purchased from Cell Signaling Technology (Danvers, MA, USA). L-TRP was dissolved in cell culture medium. AA was dissolved in 1 N NaOH, and then in phosphate-buffered saline (PBS) (the maximum pH difference in the tested concentration range was 0.1). CBA and vemurafenib were dissolved in dimethyl sulfoxide. Dilutions of the tested substances added to the cell cultures were prepared in the culture medium.

### 4.2. Cell Culture

Human melanoma A375 (CRL-1619, passage number 7-21) and SK-MEL-3 (HTB-69, passage number 17-19) cells were obtained from the American Type Culture Collection (ATCC; Manassas, VA, USA). Human melanoma G361 (88030401, passage number 11-13) cells were obtained from the European Collection of Authenticated Cell Cultures (ECACC; Porton Down, Salisbury, UK). A375 cells were maintained in Dulbecco’s modified Eagle’s medium supplemented with 10% fetal bovine serum (FBS) and antibiotics (penicillin (100 U/mL) and streptomycin (100 µg/mL)). G361 and SK-MEL-3 cells were cultured in McCoy’s 5A modified medium supplemented with 15% FBS and antibiotics. Peripheral blood acute monocytic leukemia THP-1 cells (ATCC, TIB-202, passage number 15-20) were used as a model of human macrophages. The cells were maintained in RPMI 1640 medium supplemented with 10% FBS and antibiotics. For differentiation into macrophage-like cells, THP-1 cells were seeded in 96-well plates at a density of 5 × 10^4^ cells per well and stimulated with 100 ng/mL phorbol 12-myristate 13-acetate (PMA; Sigma-Aldrich) for 48 h. Following differentiation, adherent cells were washed with culture medium and further incubated for 24 h in fresh PMA-free medium to obtain resting macrophages (M0). Cell cultures were maintained in a humidified atmosphere of 95% air and 5% CO_2_ at 37 °C (standard conditions). Cell culture reagents were obtained from Merck. Cell lines were routinely checked for *Mycoplasma* contamination using Hoechst 33342 staining.

### 4.3. Preparation of Conditioned Medium (CM)

Human melanoma A375 cells were seeded into 6-well plates at the density of 6  ×  10^4^ cells/well. The next day, cells were exposed to fresh culture medium (control) or tested compounds: vemurafenib (10 µM), TRP (1 mM), AA (1 mM), CBA (100 µM), or combinations of TRP, AA, or CBA with vemurafenib. Cells were incubated for 24 h in standard conditions. Cell-culture supernatants were collected, centrifuged at 4 °C for 10 min, and stored at −80 °C for further experiments.

### 4.4. MTT Assay

A375, G361, and SK-MEL-3 cells were plated in 96-well microplates at a density of 1 × 10^4^ cells/well (Thermo Fisher Scientific, Nunc, Roskilde, Denmark). The next day, melanoma cells were exposed to fresh cell culture medium (control) or serial dilutions of tested compounds (TRP, AA: 1 pM, 1 nM, 1 µM, 1 mM, 5 mM; CBA: 1 pM, 1 nM, 1 µM, 100 µM). The range of tested concentrations was determined in preliminary studies, and the maximum concentration resulted from the solubility of the tested substances and the limitation of non-specific effects of solvents. Cells were incubated for 24 or 96 h in standard conditions. In another experimental scheme, A375 cells were exposed to fresh cell culture medium (control), TRP (1 mM), its metabolites (AA: 1 mM; CBA: 100 µM), vemurafenib (10 µM), or combinations of TRP, AA, or CBA with vemurafenib. The cells were incubated for 24 h in standard conditions. MTT assay was also used to determine the cell viability of THP-1-derived macrophages. THP-1-derived macrophages cultured in 96-well plates were treated with the indicated concentrations of the examined substances for 24 h or with conditioned medium from tumor cells (CM, 50%) for 24, 48, or 96 h. Following treatment, MTT reagent (3-(4,5-dimethylthiazol-2-yl)-2,5-diphenyltetrazolium bromide; Merck) prepared as a 5 mg/ml stock solution in PBS was added to each well. Cells were then incubated for 3 h at 37 °C in a humidified atmosphere containing 5% CO_2_. Subsequently, lysis buffer (10% SDS in 0.01 M HCl) was added to each well, and cells were incubated for an additional 24 h to dissolve the formed formazan crystals. Absorbance was measured at 570 nm using a microplate reader, according to the procedure described in the previous paper [3].

### 4.5. Migration Assay

The effect of tested compounds on melanoma A375 cell motility was assessed by the scratch assay as previously described [57]. A375 cells were plated at 1 × 10^5^ cells on 6-well plates (Thermo Fisher Scientific. The next day, the cell monolayer was scratched with a sterile pipette tip and rinsed with PBS to remove cell debris. Cells were exposed to culture medium (control) or serial dilutions of tested compounds (TRP, AA: 1 µM, 1 mM, 5 mM; CBA: 1 µM, 100 µM) for 24 h. Then, cells were stained with the May–Grünwald–Giemsa method. Cells migrated to the wound area were counted using an Olympus CKX53 microscope with CellSens Standard 2.3 software (Olympus, Tokyo, Japan).

### 4.6. Western Blotting

A375 melanoma cells were exposed to fresh culture medium (control) or serial dilutions of TRP, AA (1 µM, 1 mM, 5 mM), or CBA (1 µM, 100 µM) for 24 h in standard conditions. The concentrations were selected based on the results of the MTT Assay. Next, cells were lysed in RIPA buffer (Thermo Fisher Scientific). The concentration of isolated proteins was determined by the BCA Protein Assay Kit (Thermo Fisher Scientific). The detailed procedure for western blotting has been described previously [3,17]. The following primary antibodies were used: anti-p21, anti-p53, anti-phospho-ERK1/2, anti-ERK1/2, anti-β-actin (1:1000; Cell Signaling Technology). Secondary anti-rabbit antibodies conjugated to horseradish peroxidase were used (1:2000; Cell Signaling Technology).

### 4.7. Assessment of Cell-Cycle Progression by Flow Cytometry

Cell-cycle progression was assessed using flow cytometry based on DNA content measurements. After 24 h of treatment, cells were collected by trypsinization, washed with PBS, and centrifuged. The resulting cell pellet was fixed in 75% cold ethanol and incubated at −20 °C for at least 24 h. After fixation, cells were centrifuged and washed with PBS to remove residual ethanol. Cells were then stained with PI/RNase Staining Buffer (Becton Dickinson, San Jose, CA, USA) following the manufacturer’s protocol. Samples were analyzed using the BD Accuri C6 Plus Flow Cytometer (BD Biosciences, San Jose, CA, USA). Data analysis began with gating to select the cell population based on forward scatter (FSC) and side scatter (SSC) parameters, thereby excluding debris and non-cellular particles. Singlet cells were then identified to remove doublets and cell aggregates. For the singlet population, DNA content was analyzed by generating histograms of propidium iodide fluorescence intensity. The proportions of cells in the G0/G1, S, and G2/M phases were determined using BD Accuri C6 Plus software.

### 4.8. Measurement of ROS and NO Production

THP-1-derived macrophages cultured in black, clear-bottom 96-well plates were incubated in the presence of the examined substances or tumor CM. To assess intracellular ROS or NO levels, cells were incubated with either 10 μM 2′,7′-dichlorodihydrofluorescein diacetate (DCF-DA; Sigma Aldrich, St. Louis, MO, USA) for ROS detection or 10 μM 4-amino-5-methylamino-2′,7′-difluorofluorescein diacetate (DAF-FM DA; Sigma Aldrich) for NO detection for 30 min at 37 °C in a humidified atmosphere containing 5% CO_2_. Fluorescence intensity was measured using a Victor X4 multilabel plate reader (PerkinElmer, Shelton, CT, USA). Measurements were performed using excitation and emission filters of 485 and 535 nm, respectively, and the results were expressed as a percentage of the control group.

### 4.9. Bioinformatic Analysis

Correlation analysis was performed by GEPIA2 (the Cancer Genome Atlas (TCGA) data for Skin Cutaneous Melanoma) and statistically analyzed by Spearman correlation (*p* < 0.05) [25,36]. Retrieved expression values are provided in transcripts per million (TPM). The STRING database version 12.0 (https://string-db.org/) was used to construct the protein–protein interaction network and the bubble plot by searching for interactions among BRAF, TRP, kynureninase (KYNU), kynurenine 3-monooxygenase (KMO), cyclin-dependent kinase inhibitor 1 (CDKN1A), and cellular tumor antigen p53 (TP53). Nodes without any interactions were removed from the input nodes.

### 4.10. Data Analysis

Data were presented as the mean value (at least three independent biological replicates) and standard deviation (SD). Statistical analysis was performed using one-way ANOVA with Tukey or Dunnett post hoc tests, or an unpaired *t*-test (*p* < 0.05 was considered statistically significant). Western blots were performed in triplicate (biological replicates). Densitometric analysis was performed using NIH ImageJ 1.54g software. The data were normalized relative to β-actin.

## Data Availability

The data that support the findings of this study are available from the corresponding author upon reasonable request.

## Supplementary Materials

The following supporting information can be downloaded at: https://www.mdpi.com/article/10.3390/ijms27219908/s1.

## Abbreviations

The following abbreviations are used in this manuscript:

AA	anthranilic acid
AADAT	aminoadipate aminotransferase
AhR	aryl hydrocarbon receptor
BRAF	B-Raf proto-oncogene, serine/threonine kinase
CBA	cinnabarinic acid
CDKN1A	cyclin-dependent kinase inhibitor 1A
CM	conditioned medium
DAF-FM DA	4-amino-5-methylamino-2′,7′-difluorofluorescein diacetate
DC	dendritic cell
DCF-DA	2′,7′-dichlorodihydrofluorescein diacetate
DMEM	Dulbecco’s modified Eagle’s medium
DMSO	dimethyl sulfoxide
FBS	fetal bovine serum
FICZ	6-formylindolo [3,2-b]carbazole
GOT2	glutamic-oxaloacetic transaminase 2
HS	hidradenitis suppurativa
IAId	indole-3-aldehyde
ICB	immune checkpoint blockers
IDO	indoleamine 2,3-dioxygenase
IL	interleukin
KP	kynurenine pathway
KAT	kynurenine aminotransferase
KYAT	kynurenine aminotransferase
KSR	kinase suppressor of ras
KYN	kynurenine
KYNA	kynurenic acid
KYNU	kynureninase
MAP2K2	Mitogen-Activated Protein Kinase Kinase 2
MAP3K11	Mitogen-Activated Protein Kinase Kinase Kinase 11
MDSCs	myeloid-derived suppressor cells
MTT	3-(4,5-dimethylthiazol-2-yl)-2,5-diphenyltetrazolium bromide
NAD	nicotinamide adenine dinucleotide
NO	nitric oxide
PBS	phosphate-buffered saline
PMA	phorbol 12-myristate 13-acetate
ROS	reactive oxygen species
SD	standard deviation
SDS	sodium dodecyl sulphate
TAM	tumor-associated macrophage
TDO2	tryptophan 2,3-dioxygenase
TNF	tumor necrosis factor
TPM	transcripts per million
TRP	tryptophan
