# Supplementary material for: Tryptophan and Its Selected Metabolites Modulate Melanoma-Cell Biology: A Preliminary In Vitro Study
Source: Int J Mol Sci. 2026 Nov 5;27(21):9908. doi: 10.3390/ijms27219908 (PMC13763768; doi:10.3390/ijms27219908)
Supplement: Supplementary file 1 [file ijms-27-09908-s001.zip › ijms-4608014-supplementary-figures.pdf]

Supplementary Data

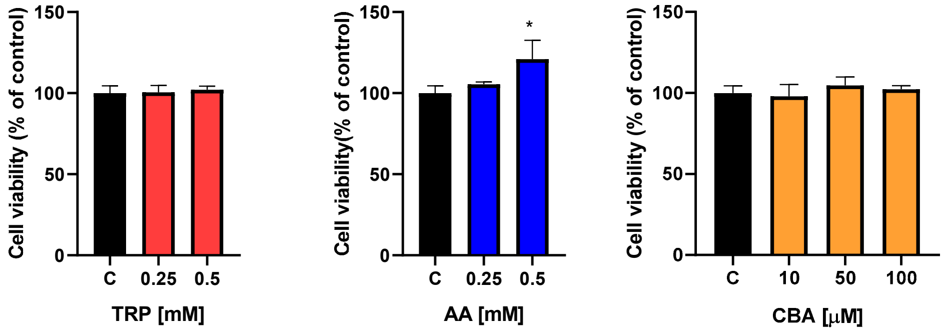

Figure S1. The effect of TRP and its selected metabolites on the viability of macrophages. PMA-induced THP-1 cells were exposed to a range of concentrations of TRP (0.25, 0.5 mM), AA (0.25, 0.5 mM), or CBA (10, 50, 100 μM) for 24 hours. The cell viability were determined by MTT Assay. Data are presented as % of control (Control – non-treated, 100%). Statistical significance (one-way ANOVA with Dunnett post hoc test, p < 0,05 (*).

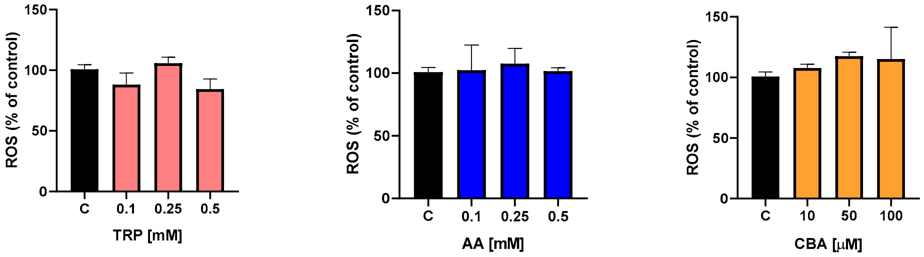

Figure S2. The effect of TRP and its selected metabolites on ROS production in macrophages. PMA-induced THP-1 cells were exposed to a range of concentrations of TRP (0.1, 0.25, 0.5 mM), AA (0.1, 0.25, 0.5 mM), or CBA (10, 50, 100 μM) for 24 hours. The intracellular ROS levels were determined by DCF-DA. Data are presented as % of control (Control – non-treated, 100%). Statistical significance (one-way ANOVA with Dunnett post hoc test, p < 0,05 (*).

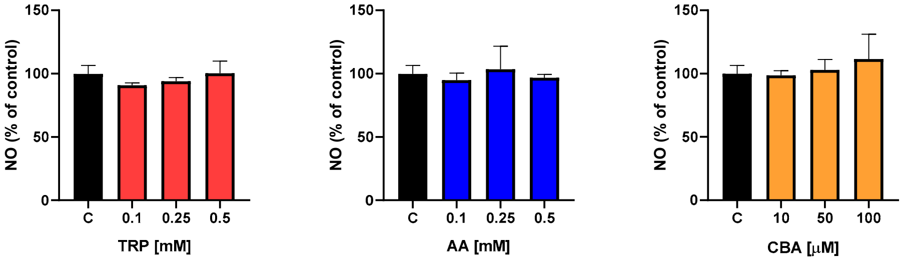

Figure S3. The effect of TRP and its selected metabolites on NO production in macrophages. PMA-induced THP-1 cells were exposed to a range of concentrations of TRP (0.1, 0.25, 0.5 mM), AA (0.1, 0.25, 0.5 mM), or CBA (10, 50, 100 μM) for 24 hours. The NO levels were determined by DAF-FM DA. Data are presented as % of control (Control – non-treated, 100%). * statistical significance (one-way ANOVA with Dunnett post hoc test, p < 0,05 (*).
